# Hypothermia after aneurysmal subarachnoid hemorrhage

**DOI:** 10.1186/cc11274

**Published:** 2012-06-07

**Authors:** Martin Seule, Emanuela Keller

**Affiliations:** 1 Neurointensive Care Unit, Department of Neurosurgery, University Hospital Zurich, Switzerland

## Introduction

Aneurysmal subarachnoid hemorrhage (SAH) is a common and frequently devastating condition, accounting for 1 to 7% of all strokes with an incidence of 9.1 per 100,000 [1]. Major advances in SAH management over the past three decades have decreased case fatality by 0.8% per year, but it is still 40% and many survivors have long-term disabilities [2]. The most important and potentially treatable complication is development of delayed cerebral ischemia (DCI), which can progress to cerebral infarction associated with poor outcome.

The pathogenesis of DCI is multifactorial and assumed to be initiated in the early phase of SAH [3]. The onset of SAH is characterized by a short-lasting and cerebral perfusion pressure (CPP)-dependent decrease in cerebral blood flow (CBF) leading to global cerebral ischemia [4]. Elevated intracranial pressure (ICP) and acute cerebral ischemia are the main factors for early disruption of the blood-brain barrier as well as impairment of autoregulation associated with brain edema and brain swelling. Important pathogenic mechanisms of CPP-independent hypoperfusion include acute vasoconstriction, cortical spreading ischemia, and activation of the inflammatory response. The release of oxyhemoglobin and endothelin-1 (ET-1) are the key factors for cortical spreading ischemia, reduced nitric oxide (NO) availability, and secondary cytotoxic edema formation. Cerebral vasospasm (CVS) is a delayed morphological narrowing of cerebral arteries, occurring 4 to 10 days after SAH. Although CVS have been associated with DCI, it is generally accepted that CVS is not solely responsible for DCI [5]. In fact, DCI may occur in the absence of CVS and *vice versa *and the distribution of CVS may fail to reliably predict the subsequent pattern of cerebral infarction [6].

Neuroprotective strategies to prevent DCI have been mainly focused on treatment of CVS, but despite extensive research, effective and/or causative prophylaxis and treatment are not available [7]. So far, oral nimodipine is the only drug that can reduce the incidence of DCI and poor outcome, but there is no beneficial effect on CVS [8]. Hypothermia (HT) treatment exerting numerous protective effects such as a decrease in cerebral metabolism [9], stabilization of the blood-brain barrier [10], reduction of cerebral edema [11], suppression of excitatory neurotransmitter concentrations [12] and inflammatory reactions [13] seems to be well suited as a neuroprotective strategy. In the following, the clinical application of HT after SAH is presented by reviewing the existing literature.

## Hypothermia during aneurysm surgery

In the past, promising studies on intraoperative HT during aneurysm surgery as an attempt to reduce ischemic injury have been published [14-18]. The Intraoperative Hypothermia for Aneurysm Surgery Trial (IHAST) applied HT in a randomized study in 1,001 patients with good-grade SAH (WFNS 1 to 3); however, it found no improvement in neurological outcome 3 months after surgery [19]. *Post-hoc *analysis demonstrated no difference in the incidence of cognitive impairment between hypothermic and normothermic groups [20]. Furthermore, there was no evidence for the benefit of intraoperative HT on 24-hour and 3-month outcome in patients who underwent temporary clipping [21]. It has to be noted that these results only apply to good-grade SAH patients and may not be extrapolated to the general SAH population. This suggests that only a carefully selected subgroup of patients, with specific complications induced by SAH, may benefit from HT treatment at a particular time and for certain duration.

## Hypothermia in patients with poor-grade subarachnoid hemorrhage

Several experimental studies have demonstrated that HT is effective in minimizing neuronal damage, if induced before or early after the SAH. Kawamura and colleagues found a reduced expression of c-jun and hsp-70 mRNA, indicating a reduced stress response that may otherwise manifest as necrosis or apoptosis [22]. Thome and colleagues and Schubert and colleagues demonstrated that early induction of HT, up to 60 minutes after SAH, reversed CPP-independent hypoperfusion and brain edema formation, preserved cerebral autoregulation, and reduced accumulation of lactate and glutamate [11,12,23]. On the other hand, delayed induction of HT, up to 180 minutes after SAH, failed to reduce brain edema formation, thus indicating a limited time window of HT application [24]. However, HT was effective in reducing ICP and associated with improved neurological recovery. These studies encourage early induction of HT in poor-grade SAH patients, for example, as soon as signs of brain swelling are seen on CT scans.

So far, only few retrospective case series have investigated the effect of mild HT during the acute phase of SAH. Table 1 presents an overview of these studies applying HT within 24 hours after SAH to patients with poor-grade SAH (WFNS 4 to 5). The duration of HT varied between 2 and 14 days. Overall outcome results were unsatisfactory with a mortality rate of 47.4% and favorable outcome in less than one-quarter of cases. Yasui and colleagues evaluated seven patients admitted within 6 hours from symptom onset and treated with HT for 48 hours [25]. PET studies during HT revealed a reduction in cerebral oxygen metabolism exceeding the decrease in CBF, thus indicating a state of luxury perfusion. However, according to the Barthel Index only two patients were independent, one was partially dependent, able to walk without assistance, and more than one-half of the patients bedridden at 12 months after SAH. Anei and colleagues compared outcome results before and after introduction of HT treatment to their institution and found no significant difference in the mortality rate with 56.3% and 57.9%, respectively [26]. The authors noted that post-HT fever can be a serious complication resulting in brain swelling and unfavorable outcome. In contrast to these studies, Gasser and colleagues showed more promising outcome results in 21 patients with poor-grade SAH (WFNS 4 to 5) and induction of HT after developing intracranial hypertension (>15 mmHg) that was refractory to conventional treatment. HT was induced on average 4.2 ± 3.3 days after SAH and maintained for 4.3 ± 3.9 days. Prolonged HT (>72 hours) was associated with an increased risk of systemic complications, but 10 patients (47.6%) showed favorable outcome (GOS 4 to 5) and five patients died (23.8%).

**Table 1:** Studies applying hypothermia on day of aneurysm rupture to patients with poor-grade SAH (WFNS 4 to 5 or H&H IV to V)

Study	Number of patients (*n*)	Target temperature (°C)	Duration of hypothermia (days)	Favorable outcome*, % (*n*)	Mortality rate, % (*n*)
Nagao and colleagues (2000)	9	32 to 34	5 to 14	44.4% (4)	55.6% (5)
Yasui and colleagues (2002)	7	33 to 34	2	42.8% (3)*	0.0% (0)
Nakamura and colleagues (2002)	3	32 to 34	5 to 14	0.0% (0)	66.7% (2)
Anei and colleagues (2010)	19	34	2	5.3% (1)	57.9% (11)
Total	38			21.1% (8)	47.4% (18)

*GOS ≥4 or mRS ≤3.

## Hypothermia in patients with delayed cerebral ischemia/cerebral vasospasm

Recently, a SAH-CVS model in dogs demonstrated that HT can attenuate the degree of CVS up to 14 days after SAH, possibly by regulating the levels of ET-1 and NO [27]. The duration of HT was directly proportional to the duration of relieving CVS. Table 2 presents an overview of clinical studies applying HT to patients with symptomatic CVS leading to DCI. Nagao and colleagues treated five patients with good-grade SAH (H&H I to III), starting HT either during delayed aneurysm clipping or if CVS was refractory to hyperdynamic and endovascular therapy [28]. Four patients survived with favorable outcome and one patient was severely disabled. In a follow-up study, Nagao and colleagues included eight patients with good-grade SAH (H&H II to III), and seven patients had favorable outcome and one survived severely disabled [29]. According to SPECT studies, HT was associated with decreased CBF levels in all patients. Nakamura and colleagues reported a reduction in arterio-jugular oxygen difference (AVDO_2_) during HT in five patients (H&H III to IV), thus indicating a reduced metabolic demand [30]. All patients received hyperdynamic therapy before and during HT, but outcome results were unsatisfactory. Possible contributing factors of poor outcome include a higher grade of SAH and it is unclear whether endovascular treatment was applied or not. In our series of 100 SAH patients treated with HT, 28 patients had symptomatic CVS refractory to hypertensive therapy and endovascular treatment [31]. HT was combined with barbiturate coma in 23 of 28 patients and maintained until CVS resolved or severe side effects occurred (mean duration 5.7 ± 3.3 days). Although the majority of patients had poor-grade SAH (H&H 4 to 5 in 57.1%, Fisher 3 to 4 in 85.7%), favorable outcome (GOS 4 to 5) was achieved in 57.1%. In patients with intracranial hypertension (>20 mmHg) with and without refractory CVS, favorable outcome was obtained in only 25.0% and 26.5%, respectively. Systemic side effects possibly caused from HT and/or barbiturate coma included pneumonia in 52.0%, thrombocytopenia (<100,000/µl) in 47.0%, septic shock syndrome in 40.0%, and acute respiratory distress syndrome in 16.0%. In a subgroup of seven patients with combined HT and barbiturate coma, daily levels of IL-6, IL-1β, TNFα, and leukocyte count in the cerebrospinal fluid and plasma were quantified [13]. IL-6 levels in the cerebrospinal fluid and systemic IL-1β levels were significantly lower compared with patients receiving barbiturate coma alone (*n *= 8), thus indicating HT-related attenuation of the inflammatory response.

**Table 2:** Studies applying hypothermia to patients with symptomatic vasospasm refractory to conventional treatment

Study	Number of patients (*n*)	Target temperature (°C)	Duration of hypothermia (days)	Favorable outcome*, % (*n*)	Mortality rate, % (*n*)
Nagao and colleagues (2000)	5	32 to 34	5 to 14	80.0% (4)	0.0% (0)
Nakamura and colleagues (2002)	5	32 to 34	5 to 14	40.0% (2)	60.0% (3)
Nagao and colleagues (2003)	8	32 to 34	5 to 12	87.5% (7)	0.0% (0)
Seule and colleagues (2009)	28	33 to 34	1 to 16	57.1% (16)	28.6% (8)
Total	46			63.0% (29)	26.1% (12)

Note: *GOS ≥4 or mRS ≤3.

## Conclusion

So far, the evidence of HT on improved outcome after SAH is limited. Intraoperative HT has been abandoned based on the randomized Intraoperative Hypothermia Study on Aneurysm Surgery in good-grade SAH patients. The available data suggest that HT may improve outcome in a carefully selected subgroup of patients developing intracranial hypertension and/or symptomatic CVS that are refractory to conventional treatment. Further evaluation of cerebral hemodynamics and oxygenation during HT treatment is required to obtain important insights in the effects of HT and to identify patients who may benefit most.
